# Identification and functional analysis of class 3 lipases associated with triacylglycerol metabolism in *Nannochloropsis oceanica* (Eustigmatophyceae)

**DOI:** 10.1111/jpy.70203

**Published:** 2026-07-09

**Authors:** Masako Iwai, Koichi Hori, Tomokazu Kurita, Kumiko Okazaki, Shinichiro Maeda, Akihide Takami, Takashi Yamamoto, Atsushi Sakamoto, Hiroyuki Ohta, Mie Shimojima

**Affiliations:** ^1^ Phytolipid Technologies Co. Ltd. Yokohama Japan; ^2^ School of Life Science and Technology Institute of Science Tokyo Yokohama Japan; ^3^ Genome Editing Innovation Center Hiroshima University Higashi‐Hiroshima Japan; ^4^ Algal Bioenergy Innovation Laboratory, Joint Research Course for Next Generation Automobile Technology, Graduate School of Integrated Sciences for Life Hiroshima University Higashi‐Hiroshima Japan; ^5^ Mazda Motor Corporation Hiroshima Japan; ^6^ Division of Integrated Sciences for Life, Graduate School of Integrated Sciences for Life Hiroshima University Higashi‐Hiroshima Japan

**Keywords:** lipase, lipid metabolism, microalgae, *Nannochloropsis*, nutrient deficiency, triacylglycerol

## Abstract

Microalgae accumulate triacylglycerols (TAGs) as storage lipids in response to nutrient limitation, yet the mechanisms governing TAG turnover remain incompletely understood. In the oleaginous microalga *Nannochloropsis oceanica*, functional studies of TAG degradation have focused on lipases containing patatin domains, whereas the roles of other lipase families remain poorly characterized. To address this shortcoming, we focused on class 3 lipases, a group distinct from patatin‐domain TAG lipases, and examined their roles in TAG metabolism in *N. oceanica*. Based on genome sequencing information and domain‐based screening, 23 putative class 3 lipases were identified and classified into two major orthogroups, including a lineage‐specific group unique to *Nannochloropsis*. Transcriptomic data obtained under nutrient‐deficient conditions were used to prioritize candidate genes for functional analysis. Targeted gene‐disruption mutants were examined under phosphate‐deficient conditions, which induce TAG accumulation while causing only relatively mild growth inhibition. Disruption of the *No3LIP6* or *No3LIP14* gene was consistently associated with increased TAG accumulation without severe growth defects, whereas other class 3 lipase mutants exhibited growth‐linked or condition‐dependent effects on TAG accumulation. In addition, disruption of selected class 3 lipases resulted in opposing changes in TAG‐associated fatty‐acid composition, suggesting differential contributions to TAG remodeling rather than uniform effects on TAG hydrolysis. Together, these results indicate that TAG turnover in *N. oceanica* grown under phosphate‐deficient conditions is associated with lipases beyond the patatin‐domain family, with a specific subset of class 3 lipases implicated in this process. This study highlights the functional relevance of non‐patatin TAG lipases in microalgal TAG metabolism.

**Abbreviations**
N: nitrogen
P: phosphorus
PCR: polymerase chain reaction
Pfam: Protein family database
RNA‐seq: RNA sequencing
TAG: triacylglycerol
TALEN: transcription activator‐like effector nuclease

## INTRODUCTION

Microalgae are photosynthetic microorganisms that can accumulate triacylglycerols (TAGs) as storage lipids in response to nutrient limitation (Hu et al., 2008; Hu & Gao, 2006; Iwai et al., 2014, 2015; Jia et al., 2015; Maltsev et al., 2023; Martin et al., 2014; Mühlroth et al., 2017; Rodolfi et al., 2009; Wang et al., 2009). Because TAG accumulation represents a broadly conserved physiological response across diverse taxa, microalgae provide a useful experimental system for investigating the regulation of lipid metabolism under nutrient stress. *Nannochloropsis* is a genus of marine microalgae known for the high production of photoautotrophic biomass rich in proteins and lipids, including ω‐3 fatty acids such as eicosapentaenoic acid (Al‐Hoqani et al., 2017; Ma et al., 2016). More recently, species of this genus have been recognized for their capacity for TAG accumulation (Hodgson et al., 1991; Hu et al., 2008; Hu & Gao, 2006; Iwai et al., 2015; Jia et al., 2015; Razzak et al., 2015; Rodolfi et al., 2009). *Nannochloropsis* species belong to the class *Eustigmatophyceae* (Maruyama et al., 1986), and multiple species and strains have been subjected to whole‐genome sequencing (Radakovits et al., 2012; Vieler et al., 2012; Wang et al., 2014). In parallel, genetic manipulation techniques, including homologous recombination and genome‐editing approaches (Ajjawi et al., 2017; Kilian et al., 2011; Kurita et al., 2020, 2022, 2023), have been developed, enabling targeted functional analyses of lipid metabolism in this genus. *Nannochloropsis* species have been reported to tolerate growth using wastewater‐derived nutrients (Biondi et al., 2013), a feature that supports their utility as experimental model systems and potential production platforms.

Triacylglycerol accumulation reflects a dynamic balance between lipid biosynthesis, storage, and degradation. In microalgae, multiple pathways involved in fatty‐acid synthesis and TAG assembly have been reported, and several of these pathways have also been characterized in *Nannochloropsis* (Jia et al., 2015; Li et al., 2014; Mühlroth et al., 2017). Increasing evidence indicates, however, that TAG turnover and remodeling are regulated processes contributing to lipid homeostasis under nutrient stress. Studies in microalgae support this view: In *Chlamydomonas reinhardtii*, for example, TAG lipase has been shown to control TAG mobilization during recovery from nitrogen starvation (Warakanont et al., 2019), whereas in the diatom *Phaeodactylum tricornutum*, downregulation of TAG lipase activity was shown to lead to increased TAG accumulation (Barka et al., 2016). Together, these studies imply a potential contribution of lipases to TAG dynamics across phylogenetically distinct microalgal groups.

Lipases comprise a diverse group of hydrolytic enzymes with distinct substrate specificities and include TAG lipases that regulate TAG turnover (Kelly & Feussner, 2016). In *Saccharomyces cerevisiae*, the patatin‐domain lipases Tgl3p, Tgl4p, and Tgl5p are involved in TAG mobilization (Athenstaedt & Daum, 2003, 2005). In *Arabidopsis thaliana*, the patatin‐domain TAG lipase SUGAR‐DEPENDENT1 (SDP1) initiates the breakdown of storage lipids during germination (Eastmond, 2006). The lipase SDP1 and its paralogue SDP1‐LIKE are responsible for 95% of TAG hydrolysis in germinating *Arabidopsis* seeds (Kelly et al., 2011). In microalgae, the patatin‐domain TAG lipases have also been implicated in TAG turnover. In *Phaeodactylum tricornutum*, downregulation of the lipase TGL1 increases TAG accumulation (Barka et al., 2016). In *Chlamydomonas reinhardtii*, LIP4 is involved in TAG degradation (Warakanont et al., 2019). In *Nannochloropsis oceanica*, several TAG lipases containing a patatin domain have been functionally characterized. The patatin‐domain TAG lipases NoTGL1 and NoTGL2, which are homologous to *A. thaliana* SDP1, were shown to modulate TAG metabolism, and disruption of both genes increased TAG accumulation under standard growth conditions (Nobusawa et al., 2019). In addition, knockout of another TAG lipase gene, NoTGL1990, increased TAG abundance (Miao et al., 2024). Despite these advances, a number of additional putative lipases have been reported in *Nannochloropsis*, including members of the class 3 lipase family, with several suggested as candidate TAG lipases based on amino‐acid sequence similarity (Kang et al., 2017; Okazaki et al., 2025; Zhang et al., 2022; Zienkiewicz et al., 2020). Class 3 lipases are members of the α/β‐hydrolase superfamily, a functionally diverse group of enzymes, and are widely distributed among animals, plants, and prokaryotes (Arpigny & Jaeger, 1999). Class 3 lipases contain the consensus GxSxG motif and possess a catalytic triad consisting of Ser‐His‐Asp/Glu in their active sites; they hydrolyze long‐chain acyl triglycerides into di‐ and monoglycerides, glycerol, and free fatty acids. One of the class 3 lipases, the acid lipase oil body lipase 1 (RcOBL1) from castor bean endosperm, hydrolyzes TAGs with broad substrate specificity but shows no activity toward phospholipids (Eastmond, 2004). In addition, oil body lipase 1 (AtOBL1) in *A. thaliana* has been reported to localize to lipid droplets in pollen tubes (Müller & Ischebeck, 2018) and is essential for pollen‐tube growth and functions as an acid lipase capable of hydrolyzing various TAGs without a clear preference for acyl chains; it can also cleave 1,2‐diacylglycerol, 1,3‐diacylglycerol, and 1‐monoacylglycerol. In contrast to patatin‐domain TAG lipases, the functional roles of class 3 lipases in TAG turnover and remodeling remain poorly characterized. Thus, how non‐patatin‐domain lipases contribute to TAG metabolism under nutrient‐deficient conditions represents a key unresolved issue.

In this study, we identified and phylogenetically classified class 3 lipases in *Nannochloropsis oceanica* NIES‐2145 using genome sequence information and domain‐based screening. Transcriptomic data obtained under nutrient‐deficient conditions were used to support candidate prioritization, and selected genes were functionally examined using targeted gene‐disruption mutants. Growth characteristics, TAG accumulation, and TAG‐associated fatty‐acid composition were analyzed under phosphate (P)‐deficient conditions, which induce TAG accumulation while causing relatively mild growth inhibition compared with nitrogen deprivation (Abida et al., 2015; Iwai et al., 2014, 2015; Rodolfi et al., 2009; Sharma et al., 2012). By focusing on class 3 lipases distinct from previously characterized patatin‐domain TAG lipases, we aimed to evaluate their potential roles in TAG turnover and remodeling and to assess the extent of functional specialization among lipases expressed by *N. oceanica*.

## MATERIALS AND METHODS

### Strains, culture conditions, and growth measurements

*Nannochloropsis oceanica* strain NIES‐2145 was obtained from the Microbial Culture Collection, the National Institute for Environmental Studies, Japan (the NIES Collection). Liquid cultures of strain NIES‐2145 were grown in 300‐mL culture flasks containing 100 mL of F2N medium (Kilian et al., 2011) with Daigo's artificial seawater SP (36 g · L^−1^; FUJIFILM Wako Pure Chemical Corporation, Japan) at 25°C under continuous white light on a reciprocal shaker (120 rpm). Cultures of *No3LIP7*, *No3LIP14*, and *No3LIP18* gene mutants were illuminated from above using fluorescent light (30–35 μmol photons · m^−2^ · s^−1^). Cultures of *No3LIP6* and *No3LIP10* gene mutants were illuminated from below using LED light (35–40 μmol photons · m^−2^ · s^−1^). Agar plates were prepared with 0.8% Bacto agar (BD Difco, United States) in F2N 50% SW medium, which is identical to F2N medium except that it contains 50% artificial seawater. A F2N 50% SW medium plate containing 2 μg · L^−1^ zeocin (Invitrogen, United States), 350 μg · L^−1^ hygromycin B (FUJIFILM Wako Pure Chemical Corporation), or 150 μg · L^−1^ paromomycin sulfate (FUJIFILM Wako Pure Chemical Corporation) was used to screen transformants (Iwai et al., 2015; Murakami et al., 2018). For nutrient deprivation, cells were collected by centrifugation and inoculated into F2N medium lacking NaH_2_PO_4_ (–P medium) after rinsing twice with a defined medium. In this study, physiological analyses of cell growth and TAG accumulation in wild‐type and mutant strains were conducted under control and P‐deficient conditions using the F2N medium system. Nitrogen (N)‐deficient conditions were not examined in this culture system, as nitrogen deprivation in *Nannochloropsis* severely inhibits growth, which complicates the quantification of TAG accumulation in gene‐disruption mutants. The cultures were inoculated with an initial cell density of 2–3 × 10^6^ cells per mL. Cell growth was evaluated by counting the number of cells using a hemocytometer and a CellDrop BF (DeNovix Inc., United States).

### RNA sequencing and transcriptome analysis under nutrient‐deficient conditions

Cells for RNA sequencing (RNA‐seq) were cultured for 0, 3, or 5 days in modified 271 medium (Satoh et al., 2013) containing 36 g · L^−1^ Daigo's artificial seawater SP, 2.5 g · L^−1^ KNO_3_, 0.25 g · L^−1^ Na_2_HPO_4_, 75 mg · L^−1^ Fe(III)‐EDTA, 1.1 mg · L^−1^ H_3_BO_4_, 9.15 mg · L^−1^ MnCl_2_·4H_2_O, 1.1 mg · L^−1^ ZnSO_4_·7H_2_O, 0.4 mg · L^−1^ CuSO_4_·5H_2_O, 0.08 mg · L^−1^ MoO3, 0.12 μg · L^−1^ vitamin B12, 0.06 μg · mL^−1^ biotin, and 12 μg · L^−1^ thiamine hydrochloride. Under P‐deficient or N‐deficient conditions, medium lacking Na_2_HPO_4_ or KNO_3_, respectively, was used. Cells were harvested by centrifugation at 3000 × *g* for 10 min at 23°C, and the resulting pellets were frozen in liquid nitrogen and stored at −80°C.

For experimentation, frozen pellets were ground to a fine powder in liquid nitrogen using a mortar and pestle with six volumes of RNA extraction buffer (0.8% SDS, 25 mM Tris–HCl pH 7.6, 25 mM MgCl_2_, and 25 mM KCl) mixed with an equal volume of acid phenol, under frozen conditions. Total RNA was extracted from the aqueous phase after three rounds of acid phenol/chloroform (1:1) extraction, followed by isopropanol precipitation. RNA was further purified using the RNeasy Plant Mini kit (Qiagen, The Netherlands). Libraries were prepared using the Illumina TruSeq RNA Sample Preparation kit v2, and 76‐bp single‐end sequencing was performed on an Illumina GAIIx platform. Reads were mapped to *Nannochloropsis oceanica* NIES‐2145 transcripts using HISAT2 (Kim et al., 2019). Read counts were normalized using the TCC package (Sun et al., 2013). Hierarchical clustering of log2 fold changes of *N. oceanica* NIES‐2145 class 3 lipase genes was performed using R v4.5.0. Procedures for genomic data acquisition are described in Appendix S1.

### Identification of class 3 lipases and orthogroup assignment

Pfam domains of all proteins of *Nannochloropsis oceanica* NIES‐2145 were predicted using the databases InterProScan v1.3 and Pfam v27.0 (Quevillon et al., 2005). Amino‐acid sequences for 10 species (Table S1) were grouped into 19,555 orthogroups using OrthoFinder v2.5.2 (Emms & Kelly, 2019). Orthogroups containing *N. oceanica* NIES‐2145 lipase genes were identified based on the presence of any of the Lipase_3 (Pfam accession number: PF01764), Lipase_GDSL (Pfam accession number: PF00657), and Lipase_GDSL_2 (Pfam accession number: PF13472) domains. From these, 11 orthogroups were selected (Table 1, Table S2), including the *N. oceanica* NIES‐2145 protein possessing any one of these lipase‐related domains.

**TABLE 1:** Orthogroups containing lipase genes of *Nannochloropsis oceanica* NIES‐2145.

Orthogroup	Number of genes										*Nannochloropsis oceanica* NIES‐2145 Locus ID
Pfam domain	Cm	Ccr	Cr	Kn	At	Esi	Ptr	Ehu	Gth	Noc	(*) not detected lipase domains
OG0000141 Lipase 3	3	6	4	5	3	6	2	8	5	10	NocV09_00200110, NocV09_00400910, NocV09_00802570, NocV09_00901180, NocV09_01201880, NocV09_01300170, NocV09_03300750, NocV09_05600180, NocV09_07400050, NocV09_02800460
OG0000178 Lipase 3	2	1	7	9	7	6	5	2	5	2	NocV09_01300810, NocV09_02101390
OG0000341 Lipase 3	0	0	0	3	2	5	3	7	8	5	NocV09_01200890, NocV09_01301610(*), NocV09_01601140(*), NocV09_02800300(*), NocV09_05300010(*)
OG0000589 Lipase GDSL2	2	1	2	1	4	1	2	8	2	3	NocV09_00900970, NocV09_01900330, NocV09_08600060
OG0000746 Lipase 3	1	1	3	2	6	3	2	1	2	2	NocV09_01301950(*), NocV09_03100650
OG0003312 Lipase 3	0	0	0	0	0	0	0	0	0	11	NocV09_00702430, NocV09_00702440(*), NocV09_00801990(*), NocV09_00901140, NocV09_01300050, NocV09_02600380, NocV09_03000300, NocV09_03100040(*), NocV09_03900440, NocV09_03900450, NocV09_12300050
OG0014741 Lipase GDSL2	0	0	1	0	0	0	0	0	0	1	NocV09_00102880
OG0019290 Lipase GDSL or Lipase GDSL2	0	0	0	0	0	0	0	0	0	2	NocV09_02500240(GDSL), NocV09_03600090(GDSL2)
OG0051562 Lipase GDSL	0	0	0	0	0	0	0	0	0	1	NocV09_00102970
OG0051624 Lipase 3	0	0	0	0	0	0	0	0	0	1	NocV09_00201200
OG0052106 Lipase GDSL2	0	0	0	0	0	0	0	0	0	1	NocV09_01200320

*Note*: The abbreviations for species in each column of “Number of genes” are as follows: Cme (*Cyanidioschyzon merolae* 10D), Ccr (*Chondrus crispus* Stackhouse), Cre (*Chlamydomonas reinhardtii* CC‐503), Kn (*Klebsormidium nitens* NIES‐2285), At (*Arabidopsis thaliana*), Esi (*Ectocarpus siliculosus* Ec32), Ptr (*Phaeodactylum tricornutum* CCAP 1055/1), Ehu (*Emiliania huxleyi* CCMP1516), Gth (*Guillardia theta* CCMP2712), and Noc (*Nannochloropsis oceanica* NIES‐2145) (Table S1).

### Phylogenetic analysis

For phylogenetic analysis of orthogroup OG0000141 (a predicted Lipase_3‐containing orthogroup identified using OrthoFinder), we included two additional bacterial lipase_3 proteins—*Bacillus anthracis* (strain Sterne; WP_001260128) and *Rippkaea orientalis* PCC 8801 (WP_012595659) as outgroups. For OG0003312, eight proteins from *Nannochloropsis gaditana* B31 (Radakovits et al., 2012) and nine proteins from *N. salina* CCMP1776 (Ohan et al., 2019) were included as homologs. In addition, eight homologs retrieved from the GenBank nonredundant protein database were included as outgroups based on similarity to the *N. oceanica* NIES‐2145 proteins in OG0003312 (a predicted Lipase_3‐containing orthogroup identified using OrthoFinder). These sequences were aligned using MAFFT version v7.222 (Katoh & Standley, 2013) with the L‐INS‐i algorithm and trimmed with trimAl v1.2 using the automatic method (Capella‐Gutiérrez et al., 2009). Phylogenetic trees were constructed with both the neighbor‐joining and maximum‐likelihood methods implemented in MEGA 12 (Kumar et al., 2024). Bootstrap support values for maximum‐likelihood trees were estimated using the adaptive bootstrap method.

### Construction of class 3 lipase gene‐disruption strains

Gene‐disruption strains of class 3 lipases were generated using either transcription activator‐like effector nuclease (TALEN)‐mediated genome editing or homologous recombination, depending on the target gene. TALEN‐mediated genome editing was applied to the *No3LIP7*, *No3LIP12*, and *No3LIP14* genes. TALEN‐binding sites were designed using TAL Effector Nucleotide Targeter 2.0 (https://tale‐nt.cac.cornell.edu; Doyle et al., 2012). Each TALEN site contained both an L‐TALEN binding sequence and an R‐TALEN binding sequence. The TALEN target sites located near the start codon and recognized by TALEs containing 16–18 repeats were selected. Two TALEN sites per target gene were designed, and the cutting activities of TALEN sites were checked using human cultured cells (HEK293T cells) as previously reported (Sakuma, Hosoi, et al., 2013; Sakuma, Ochiai, et al., 2013; data not shown). The higher active TALEN site was selected for gene disruption. The TALE repeats were inserted into the L‐TALEN expression vectors or R‐TALEN expression vectors using Golden Gate assembly systems. The fragments containing the R‐TALEN expression cassette and a kanamycin resistance gene‐expression cassette were cut out from the R‐TALEN expression vectors. The resultant fragments were inserted into L‐TALEN expression vectors containing an ampicillin resistance cassette using *Bam*HI and *Eco*RI restriction enzymes. The transformant colonies containing all‐in‐one TALEN vectors were selected by using ampicillin and kanamycin double‐selection because this construction is much more difficult than using ordinary cloning. The resultant all‐in‐one TALEN vectors for targeting the *No3LIP14* gene were constructed as previously reported (Kurita et al., 2020) and did not contain CEN/ARS, and all‐in‐one ARS‐TALEN vectors for targeting the *No3LIP7* and *No3LIP12* genes were constructed as previously reported (Kurita et al., 2022). The TALEN constructs were introduced into *Nannochloropsis oceanica* cells by electroporation using either a Gene Pulser II (Bio‐Rad Laboratories, United States) or an ELEPO21 electroporator (NEPAGENE, Japan), following established protocols. Emergent colonies were screened by polymerase chain reaction (PCR) amplification of the target loci using gene‐specific primers, followed by direct sequencing to identify mutations. Frameshift mutations were successfully obtained for the *No3LIP7* and *No3LIP14* genes (Figure S1). In contrast, despite multiple attempts, only in‐frame mutations were recovered for the *No3LIP12* gene, and no frameshift knockout strains were obtained for this gene. Homologous recombination was used to generate gene‐disruption strains for the *No3LIP6*, *No3LIP10*, and *No3LIP18* genes. General strategies for producing gene‐disruption strains were performed as described previously (Iwai et al., 2015; Murakami et al., 2018, 2020; Nobusawa et al., 2017). Transformation experiments were performed using a construct developed for the nuclear transformation of *Nannochloropsis*: pMD20 with a NT7 selection cassette conferring zeocin resistance (Kilian et al., 2011). The *Streptoalloteichus hindustanus* bleomycin resistance gene (*Sh ble*) was replaced with the *Streptomyces rimosus* aminoglycoside 3‐phosphotransferase gene (*Aph8*) to confer paromomycin resistance. To make gene‐disruption constructs, ~1–2 kbp genomic fragments upstream (left flank in Figure S2) and downstream (right flank in Figure S2) of the target region were amplified by PCR using *N. oceanica* genomic DNA as templates. The resultant PCR fragments were cloned into the pMD20 vector on either side of an antibiotic resistance cassette for homologous recombination using an In‐Fusion HD Cloning Kit (TaKaRa, Japan). The resulting constructs were introduced into *N. oceanica* cells by electroporation, and stable transformants were selected on antibiotic‐containing agar plates. Transformation of algal cells with these plasmids and selection of zeocin‐ or paromomycin‐resistant stable transformants were performed as described in Murakami et al. (2018). Correct integration of the resistance cassette and disruption of the target loci were confirmed by PCR‐based genotyping (Figure S2). Primer sequences used for TALEN screening and homologous recombination are listed in Table S3.

### Lipid extraction, triacylglycerol quantification, and triacylglycerol fatty‐acid composition

Total lipids were extracted based on the method of Bligh and Dyer (1959). Triacylglycerols were separated by thin‐layer chromatography using silica gel 60 plates (Merck, Germany) as previously reported (Iwai et al., 2015). Lipids were visualized under UV light after spraying with 0.01% (w/v) primuline (Tokyo Chemical Industry, Japan) in 80% aqueous acetone. Each TAG spot was scraped from the plate and subjected to methanolysis after adding heneicosanoic acid as an internal standard. Calibration curve solutions were made with a fatty‐acid methyl ester mixture (GLC462, Nu‐Chek Prep, United States). The resultant fatty‐acid methyl ester mixtures were extracted with hexane and analyzed by gas chromatography with a flame ionization detector (GC‐2030; Shimadzu Corporation, Japan) equipped with a ULBON HR‐SS‐10 capillary column (length, 25 m; internal diameter, 0.25 mm; Shinwa Chemical Industries, Japan). Nitrogen was used as carrier and make‐up gas. The injection volume was 1 μL with a split ratio of 1:50 (Shimojo et al., 2023). Triacylglycerol amounts were expressed on both a culture‐volume basis and a per‐cell basis, consistent with the study design. Fatty‐acid composition was determined from TAG fractions and reported as relative mol% of total TAG‐associated fatty acids.

## RESULTS

### Identification and phylogenetic analysis of class 3 lipases in *Nannochloropsis*

To obtain an overview of class 3 lipases in *Nannochloropsis oceanica* NIES‐2145, the genome of this strain was sequenced and annotated in this study, with details of the sequencing and assembly procedures described in Appendix S1. Genome assembly status, gene prediction status, and comparative analyses with other *N. oceanica* strains are presented in Table S4. Conserved lipase_3 (PF01764) domain profiles were used to screen the predicted proteome, resulting in the identification of 23 genes encoding putative class 3 lipases, designated *No3LIP1* to *No3LIP23*. These candidate proteins exhibited substantial variation in predicted length, sequence similarity, and domain architecture, suggesting functional heterogeneity within the family (Figure S3).

Phylogenetic analysis incorporating representative lipase 3 homologs from algae, plants, and bacteria resolved the No3LIP proteins into two major orthogroups (Table 1), including a broadly conserved orthogroup (OG0000141, orthogroup identified using OrthoFinder; Figure S4) and a *Nannochloropsis*‐specific orthogroup (OG0003312, orthogroup identified using OrthoFinder; Figure 1). The *Nannochloropsis*‐specific orthogroup OG0003312 contained multiple class 3 lipase genes that were exclusive to *Nannochloropsis* species. Phylogenetic analysis including homologs from different *Nannochloropsis* lineages showed that each OG0003312 gene generally had orthologs in *N. gaditana* and *N. salina*. Detailed information on gene identifiers and orthogroup assignments of the identified class 3 lipases is provided in Tables S1 and S2. Together, these analyses provided a classification framework for class 3 lipases in *N. oceanica* and informed the selection of candidate genes for subsequent functional analyses.

**FIGURE 1:**
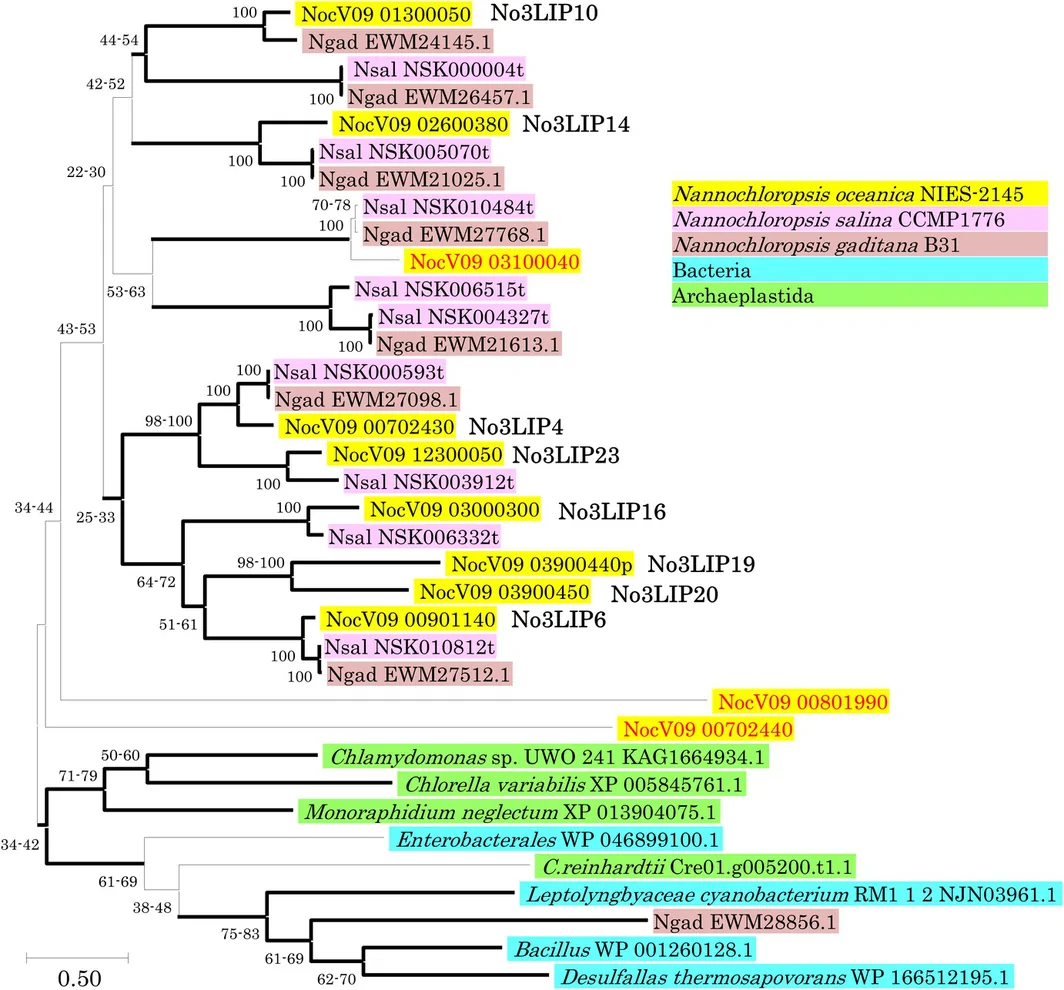
Phylogenetic tree of orthogroup OG0003312 including lipase 3 genes of *Nannochloropsis* and homologs from other algal species. The tree includes proteins from *Nannochloropsis gaditana* B31, *N. salina* CCMP1776, other Archaeplastida, and bacterial lipase 3 homologs. The tree was constructed using the maximum‐likelihood method with the LG + G (5 categories) + I model. Bootstrap support values were estimated with the adaptive bootstrap method. The scale bar indicates 0.5 amino‐acid substitutions per site. Thick branches indicate nodes with identical topologies in both the maximum‐likelihood and neighbor‐joining trees. Proteins of *N. oceanica* NIES‐2145 are shown in red text; they lack the lipase_3 pfam domain (PF01764). The GenBank accession number or protein ID is indicated for each sequence.

### Transcriptional responses of class 3 lipase genes under nutrient‐deficient conditions

In *Nannochloropsis*, TAG abundance increases significantly under P‐ or N‐deficient conditions (Hu & Gao, 2006; Iwai et al., 2015; Jia et al., 2015; Martin et al., 2014; Mühlroth et al., 2017; Rodolfi et al., 2009). To obtain an overview of how class 3 lipase genes respond to TAG‐inducing conditions, RNA‐seq data obtained under P‐ or N‐deficient conditions were examined. The expression of several *No3LIP* genes, including *No3LIP6* and *No3LIP14*, was reduced under nutrient deficiency relative to control conditions (Figure 2). Other class 3 lipase genes displayed variable or condition‐dependent transcriptional responses. Because nutrient deprivation broadly affects cellular metabolism, these transcriptional changes were utilized as a screening criterion rather than being interpreted as evidence of direct regulatory roles in TAG turnover.

**FIGURE 2:**
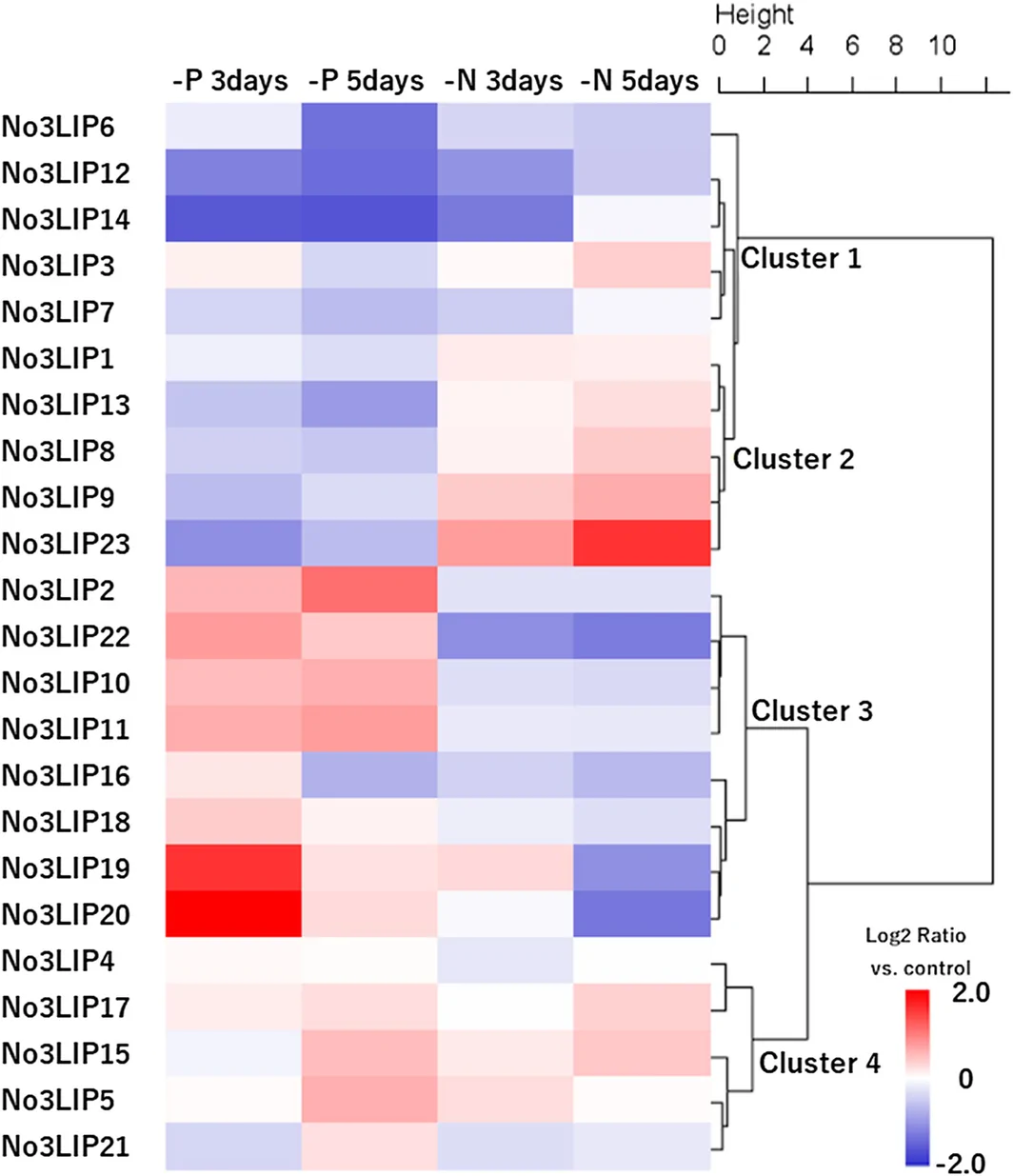
Expression profiles of Lipase_3 genes under normal, phosphate (P)‐deficient, or nitrogen (N)‐deficient conditions. *N. oceanica* NIES‐2145 was grown for 3 or 5 days under normal, phosphate (P)‐deficient, or nitrogen (N)‐deficient conditions. The heatmap shows log_2_ fold changes under nutrient‐deficient conditions relative to the corresponding control samples at each time point. Hierarchical clustering was performed using Ward's method with Pearson distance.

Although transcriptomic data were obtained under both N‐ and P‐deficient conditions, subsequent physiological and lipid analyses were conducted exclusively under P‐deficient conditions. This experimental focus reflects previous observations that N deficiency severely inhibits the growth of *Nannochloropsis*, complicating the quantification of TAG accumulation in gene‐disruption mutants; in contrast, P deficiency induces TAG accumulation with relatively mild growth impairment (Abida et al., 2015; Iwai et al., 2014, 2015; Rodolfi et al., 2009; Sharma et al., 2012). Accordingly, RNA‐seq data were used solely to assist in narrowing down candidate genes for functional analysis, and all phenotypic conclusions in this study were based on genetic and physiological experiments conducted under P‐deficient conditions.

### Growth characteristics of *No3LIP*14 gene mutants under control and P‐deficient conditions

Considering both phylogenetic classification and transcriptional responses under nutrient‐deficient conditions, the *No3LIP14* gene was selected for further phenotypic characterization to assess its potential involvement in TAG metabolism under P‐deficient conditions. Under control conditions, *No3LIP14*‐mutant strains showed slightly reduced growth compared with the wild type throughout the cultivation period (Figure 3). This reduction was modest and did not lead to a collapse of culture growth. Under P‐deficient conditions, the *No3LIP14* gene mutants exhibited growth curves similar to those of the wild type, with no pronounced differences in overall growth trends during the cultivation period. These observations indicated that disruption of the *No3LIP14* gene did not severely affect growth under control or P‐deficient conditions, allowing subsequent analyses of TAG accumulation to be interpreted without major confounding effects arising from differences in proliferation.

**FIGURE 3:**
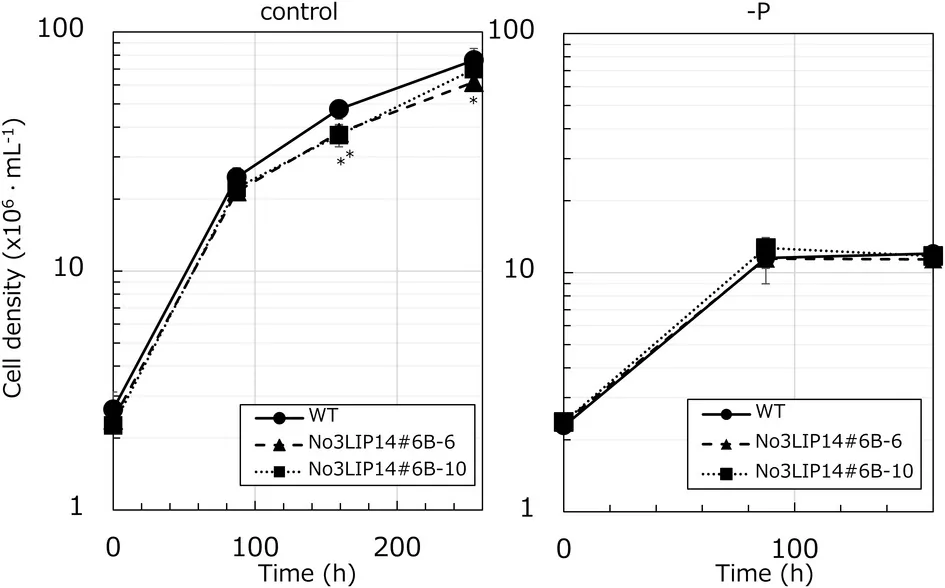
Time course for the growth of wild‐type and mutant *No3LIP14* cells under control or phosphate‐deficient conditions. Time course of cell density was monitored for cells grown in control (left column) or –P (right column) medium. WT, wild type. Data represent the mean ± *SD* of four biological replicates. Each asterisk indicates a significant difference from wild type based on Dunnett's test (*p* < 0.05).

### Effects of *No3LIP14* gene disruption on triacylglycerol accumulation under P‐deficient conditions

Triacylglycerol content was quantified in the wild‐type and *No3LIP14* gene‐mutant strains grown under control or P‐deficient conditions. Comparison of the wild type between the control and P‐deficient conditions showed that TAG accumulation increased under P deficiency on both a culture‐volume and per‐cell basis, consistent with previous observations that P limitation promotes TAG accumulation in *Nannochloropsis* (Figure 4). Under control conditions, TAG levels in the *No3LIP14* mutants were comparable to those in the wild type. Under P‐deficient conditions, the *No3LIP14* mutants accumulated higher levels of TAG than the wild type when assessed on both a culture‐volume and per‐cell basis. The level of TAG accumulation varied over the cultivation period but was consistently observed. These results indicate that disruption of the *No3LIP14* gene is associated with increased TAG accumulation under P‐deficient conditions, suggesting a potential involvement in TAG turnover during P deprivation.

**FIGURE 4:**
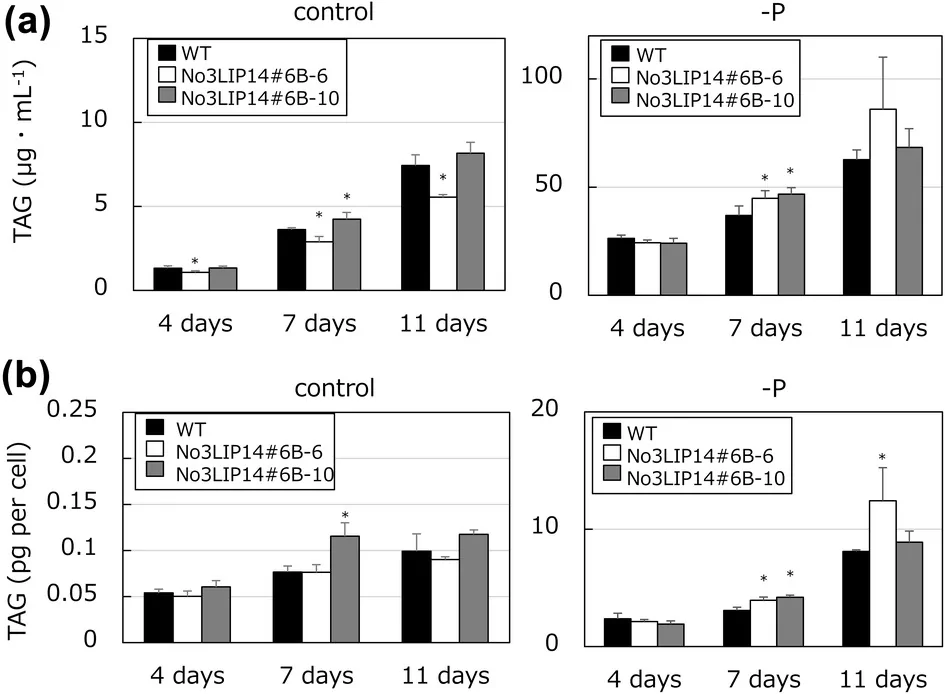
Changes in triacylglycerol content of wild type and the *No3LIP14* mutant under control or phosphate‐deficient conditions. Triacylglycerol content per culture (a) and TAG content per cell (b) were monitored for cells grown in control (left column) and –P (right column) medium for the indicated number of days. WT, wild type. Data represent the mean ± SD of four biological replicates. Each asterisk indicates a significant difference from wild type based on Dunnett's test (*p* < 0.05).

### Triacylglycerol accumulation phenotypes of additional class 3 lipase mutants

To further assess whether the TAG accumulation phenotype observed for the *No3LIP14* mutant was shared by other class 3 lipases, additional lipase mutants were analyzed under control and P‐deficient conditions. In addition to the *No3LIP14* gene, we focused on genes assigned to cluster 1 in Figure 2, which showed reduced expression under nutrient‐deficient conditions, including the *No3LIP6*, *No3LIP7*, and *No3LIP12* genes. In contrast, the *No3LIP10* and *No3LIP18* genes were assigned to cluster 3 and showed increased expression under P‐deficient conditions but decreased expression under N‐deficient conditions. Notably, the *No3LIP10* gene was included in the *Nannochloropsis*‐specific orthogroup OG0003312 (Figure 1), similar to the *No3LIP6* and *No3LIP14* genes, whereas the *No3LIP18* gene was assigned to the broadly conserved orthogroup OG0000141 (Figure S4), similar to the *No3LIP7*, and has been proposed as a candidate TAG lipase based on amino‐acid sequence similarity in previous studies (Kang et al., 2017; Zhang et al., 2022; Zienkiewicz et al., 2020). Based on these expression patterns and phylogenetic classifications, we selected the *No3LIP6*, *No3LIP7*, *No3LIP10*, *No3LIP12*, and *No3LIP18* genes for further analysis.

Under P‐deficient conditions, the *No3LIP6* mutants accumulated higher levels of TAG than the wild type during the late phase of cultivation, as assessed on both a culture‐volume and per‐cell basis (Figure 5). The growth of the *No3LIP6* mutants under P‐deficient conditions did not differ significantly from that of the wild type, indicating that enhanced TAG accumulation was not accompanied by detectable changes in proliferation.

**FIGURE 5:**
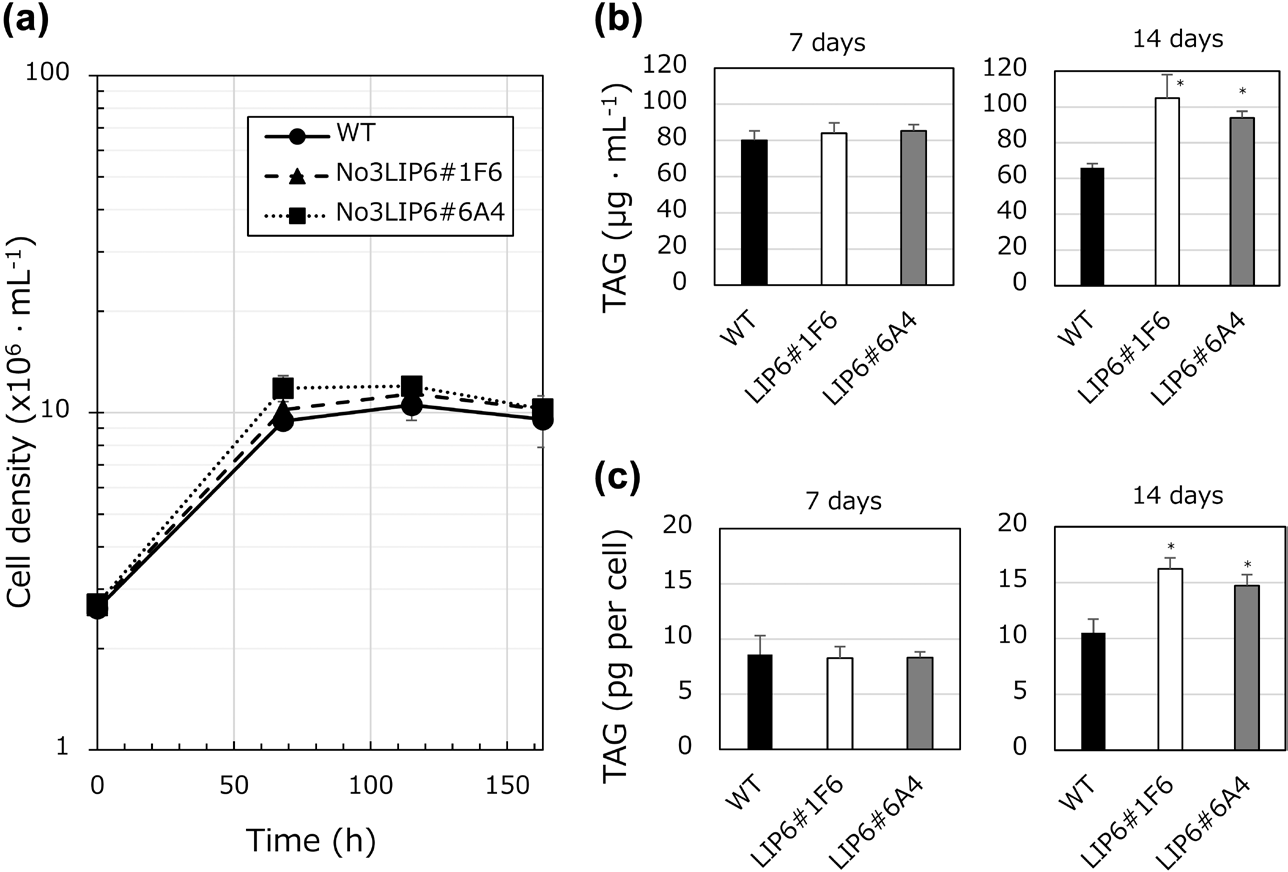
Cell density and TAG content of wild type and the *No3LIP6* mutant under phosphate‐deficient conditions. Time course for each of cell density (a), TAG content per culture (b), and TAG content per cell (c) was monitored in –P medium. WT, wild type. Data represent the mean ± *SD* of four biological replicates. Each asterisk indicates a significant difference from wild type based on Dunnett's test (*p* < 0.05).

In contrast, the *No3LIP7* mutants exhibited nutrient‐dependent differences in both growth and TAG accumulation (Figure S5). Under control culture conditions, the *No3LIP7* mutants showed slightly reduced growth compared to the wild type, whereas TAG accumulation was consistently higher than that of the wild type throughout the cultivation period. Conversely, under P‐deficient conditions, growth of the *No3LIP7* mutants was enhanced relative to that of the wild type, whereas the increase in TAG accumulation was transient and not maintained at later time points.

The growth of the *No3LIP10* mutants under P‐deficient conditions did not differ significantly from that of the wild type. For the *No3LIP10* mutants, TAG accumulation did not increase compared with the wild type under P‐deficient conditions (Figure S6). Similarly, the *No3LIP18* mutants did not exhibit increases in TAG content relative to the wild type under P‐deficient conditions (Figure S7). Attempts to generate *No3LIP12* mutant strains were unsuccessful, and therefore phenotypic analysis of this gene could not be performed.

Together, these results indicate that the *No3LIP14* and *No3LIP6* genes represent a subset of class 3 lipases for which disruption is associated with enhanced TAG accumulation under P‐deficient conditions without severe growth penalties, whereas other class 3 lipases analyzed here displayed growth‐linked, condition‐dependent, or non‐reproducible effects on TAG accumulation. This pattern supports the idea of partial functional specialization within the class 3 lipase family in *Nannochloropsis oceanica*.

### Fatty‐acid composition of triacylglycerol fractions in lipase mutants

In addition to TAG quantity, the fatty‐acid composition of TAG fractions isolated from wild‐type and lipase mutant strains was analyzed under P‐deficient conditions. Disruption of the *No3LIP14* gene was associated with statistically significant changes in the relative abundance of several major fatty acids within TAG fractions (Figure 6). Specifically, the *No3LIP14* mutant exhibited a relative decrease in monounsaturated fatty acids, including C18:1 and C16:1, accompanied by an increase in saturated C18:0 and C16:0 compared with the wild type.

**FIGURE 6:**
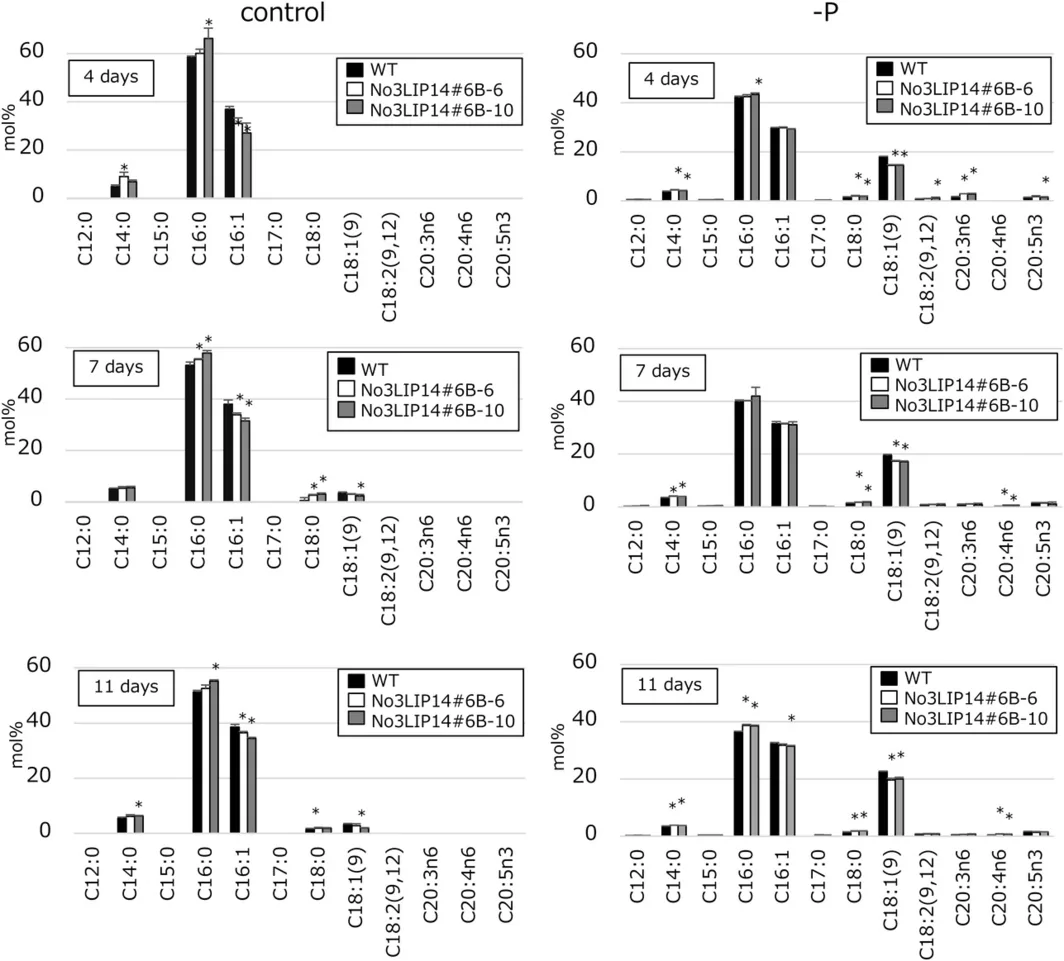
Analysis of fatty‐acid composition of the TAG fractions isolated from wild type and the *No3LIP14* mutant. The TAG fractions were isolated from cells cultured in control (left column) or –P (right column) medium for the indicated number of days. WT, wild type. Data represent the mean ± *SD* of four biological replicates. Each asterisk indicates a significant difference from wild type based on Dunnett's test (*p* < 0.05).

In contrast, disruption of the *No3LIP6* gene resulted in an opposing pattern, characterized by a relative decrease in C16:0 and concomitant increases in C18:1 and C16:1, indicating that different class 3 lipases contribute distinctively to TAG‐associated fatty‐acid composition (Figure S8). For the *No3LIP7* mutant, statistically significant shifts in fatty‐acid composition were also observed under specific conditions, including an increase in C16:0 and decreases in C16:1 and C18:1, suggesting a condition‐dependent effect on TAG fatty‐acid profiles (Figure S9). Other lipase mutants did not show consistent or statistically significant alterations in fatty‐acid composition under the conditions tested (Figures S8 and S10). Collectively, these results indicated that disruption of selected class 3 lipases influences both TAG accumulation and TAG‐associated fatty‐acid composition, with the *No3LIP14* and *No3LIP6* genes exhibiting distinct and opposing trends and the *No3LIP7* gene contributing condition‐dependent but statistically supported effects under P‐deficient conditions.

## DISCUSSION

In this work, we systematically examined 23 candidate genes belonging to the class 3 lipase family in the oleaginous microalga *Nannochloropsis oceanica* NIES‐2145, rather than focusing on previously characterized TAG lipases containing patatin domains (Miao et al., 2024; Nobusawa et al., 2019). Genetic evidence, including increased TAG accumulation in our knockout mutants, suggests that a subset of class 3 lipases may function in TAG turnover, although direct biochemical evidence demonstrating TAG hydrolysis by class 3 lipases remains limited. By integrating domain‐based identification of class 3 lipase candidates, phylogenetic analysis, transcriptional profiling under nutrient stress, and comparative analysis of targeted mutants, this work provides a thorough assessment of functional heterogeneity within this lipase family. Consequently, our results help to clarify the roles of class 3 lipases in TAG metabolism.

Phylogenetic analysis revealed that class 3 lipases in *Nannochloropsis oceanica* are distributed across two major orthogroups, including a lineage‐specific group (OG0003312) unique to *Nannochloropsis*. Each lipase 3 gene in OG0003312 generally had orthologs in *N. gaditana* and *N. salina*, suggesting that these genes diversified after the divergence of eustigmatophytes but prior to the diversification of *Nannochloropsis*. Most of the *No3LIP* genes (seven of eight) in OG0003312 were predicted to retain secretory signal peptides (Figure S3), suggesting that they may be transported via the endoplasmic reticulum to sites involved in lipid metabolism. In contrast, the *No3LIP* genes belonging to OG0000141 appear to have diversified during the evolution of primary photosynthetic eukaryotes and exhibit a wide range of domain architectures. Therefore, it remains difficult to directly associate orthogroup classification with functional specialization in this group. Under nutrient deprivation, microalgae redirect excess carbon into storage compounds such as starch and TAGs, although the preferred form varies among species. For example, *Chlamydomonas reinhardtii* accumulates both starch and TAGs under N starvation (Fan et al., 2012; Siaut et al., 2011), whereas *Phaeodactylum tricornutum* accumulates carbohydrates and lipids (Longworth et al., 2016). Under P‐deficient conditions, in which N is present in relative excess, various microalgal species accumulate excess carbon as starch or TAGs (Iwai et al., 2014, 2015; Maltsev et al., 2023; Mayers et al., 2014; Zhu et al., 2015).

Among microalgae, *Nannochloropsis* is characterized by a relatively high TAG content (Rodolfi et al., 2009), and TAG biosynthesis is strongly induced de novo under nutrient deprivation, functioning as a major carbon sink (Li et al., 2014; Zienkiewicz et al., 2020). Because TAGs serve as a primary reservoir for excess carbon, their turnover and remodeling are likely important for metabolic flexibility and recovery when nutrient availability changes. The expansion and diversification of class 3 lipases, including the *Nannochloropsis*‐specific orthogroup identified in this study, may therefore be linked to the prominent TAG turnover characteristic of this genus. The phylogenetic framework established here was used to organize candidate genes and guide functional analyses, without directly assigning enzymatic activity.

Based on phylogenetic placement and transcriptional responses under P‐ or N‐deficient conditions, five genes (*No3LIP6*, *No3LIP7*, *No3LIP10*, *No3LIP12*, and *No3LIP14*) were selected for analysis in this study. In addition, the *No3LIP18* gene, previously proposed as a TAG lipase candidate based on amino‐acid sequence similarity in previous studies (Kang et al., 2017; Zhang et al., 2022; Zienkiewicz et al., 2020), was included for comparative analysis. Transcriptional changes under nutrient stress were interpreted conservatively as indicators of potential relevance rather than as direct evidence of regulatory control. Therefore, our subsequent functional analyses focused on P‐deficient conditions, under which TAG accumulation could be evaluated with relatively little effect on growth.

Among the gene‐disruption mutants generated in this study, disruption of the *No3LIP6* and *No3LIP14* genes was consistently associated with enhanced TAG accumulation under P‐deficient conditions. This increase in TAG accumulation occurred without pronounced growth impairment, suggesting that these lipases are associated with TAG turnover and may contribute redundantly to this process.

In contrast, other class 3 lipase mutants exhibited more complex or condition‐dependent phenotypes. The *No3LIP7* mutants showed increased TAG accumulation relative to the wild type under control conditions, despite reduced growth, whereas under P‐deficient conditions, growth was enhanced but TAG accumulation increased only transiently. These observations indicate that the contribution of the *No3LIP7* gene to TAG metabolism may be linked to growth states or broader metabolic changes. In comparison, the *No3LIP10* and *No3LIP18* mutants did not exhibit increases in TAG accumulation under P‐deficient conditions and showed growth characteristics similar to those of the wild type, suggesting limited or context‐dependent involvement in TAG turnover under these conditions. The inability to obtain the *No3LIP12* gene knockout mutants suggests that this gene may play an essential role in cellular growth or viability. A summary of the mutation methods and phenotypic outcomes for analyzed class 3 lipases is provided in Table 2.

**TABLE 2:** Summary of mutant phenotypes under –P conditions.

Orthogroup	Expression profile	Gene name	Mutation method	Gene‐disruption	Growth effects	TAG levels	Fatty acid composition changes
OG0003312	Cluster 1	No3LIP6	Homologous recombination	Complete	No significant change	Increased from early culture phase	Decreased in C16:0; increased in C18:1 and C16:1
OG0000178	Cluster 1	No3LIP12	Genome editing	Not obtained	ー	ー	ー
OG0003312	Cluster 1	No3LIP14	Genome editing	Complete	No significant change	Increased in late culture phase	Decreased in C16:1 and C18:1; increased in C18:0 and C16:0
OG0000141	Cluster 1	No3LIP7	Genome editing	Complete	Increase	Transient increased	Decreased in C16:1 and C18:1; increased in C18:0
OG0003312	Cluster 3	No3LIP10	Homologous recombination	Complete	No significant change	No significant change	No significant change
OG0000141	Cluster 3	No3LIP18	Homologous recombination	Complete	No significant change	Slightly decreased	Decreased in C16:1 and C18:1; increased in C18:0 and C16:0

*Note*: Phenotypes were evaluated under phosphate‐deficient (–P) conditions. Growth effects, TAG levels, and fatty acid composition are relative to the wild type. Fatty acid composition changes refer to TAG fractions.

Taken together, these results indicate that a subset of class 3 lipases, notably the *No3LIP6* and *No3LIP14* genes, is genetically associated with changes in TAG accumulation under P‐deficient conditions. This pattern supports partial functional specialization within the class 3 lipase family rather than broad functional redundancy.

In addition to quantitative changes in TAG accumulation, disruption of specific class 3 lipases influenced TAG‐associated fatty‐acid composition. Notably, disruption of the *No3LIP14* or *No3LIP6* genes resulted in opposing shifts in the relative abundance of saturated and monounsaturated fatty acids within TAG fractions. Under P‐deficient conditions, the *No3LIP14* gene disruption was associated with decreased proportions of unsaturated fatty acids and a relative increase in saturated fatty acids, whereas the *No3LIP6* gene disruption showed the opposite trend. These contrasting patterns suggest that individual class 3 lipases may differentially influence TAG remodeling, potentially reflecting differences in substrate specificity or roles in acyl‐group turnover rather than global effects on TAG hydrolysis. Although the functions of class 3 lipases in TAG metabolism have been poorly characterized, previous results suggest that some members of this family can act on TAGs. For example, the class 3 lipase RcOBL1 from castor bean endosperm hydrolyzes TAG with broad substrate specificity (Eastmond, 2004), and AtOBL1 has been reported to function as a lipase capable of hydrolyzing various TAGs (Müller & Ischebeck, 2018). In view of these observations, the gene‐specific and opposing effects on TAG‐associated fatty‐acid composition observed in this study suggest that individual class 3 lipases in *Nannochloropsis oceanica* may play distinct roles in TAG remodeling under P‐deficient conditions.

From a practical perspective, the relative decrease in fatty‐acid unsaturation observed in the *No3LIP14* mutant may be advantageous for certain industrial applications, as increased saturation is often associated with improved oxidative stability of storage lipids. Although our results do not directly assess fuel or material properties, these compositional changes suggest that targeted manipulation of specific lipases could influence both TAG quantity and quality in future biotechnological applications.

The novelty of this study lies in its focused and comparative analysis of class 3 lipases that are distinct from previously characterized patatin‐domain TAG lipases, demonstrating that TAG turnover under P‐deficient conditions is associated with a specific subset of lipase family members rather than a single dominant enzyme. A limitation of the present work is that functional interpretations are restricted to P‐deficient conditions and are based on genetic associations rather than direct biochemical characterization of lipase activity. Nevertheless, the framework established here provides a foundation for future studies integrating biochemical analyses and multi‐gene perturbations to further elucidate the roles of class 3 lipases in microalgal TAG metabolism.

## CONCLUSIONS

This study demonstrates that class 3 lipases, which are distinct from patatin‐domain TAG lipases, are associated with changes in TAG accumulation and may contribute to TAG turnover in *N. oceanica* NIES‐2145. Disruption of the *No3LIP14* or *No3LIP6* gene was associated with enhanced TAG accumulation under P‐deficient conditions without severe growth defects, whereas other class 3 lipases showed limited or condition‐dependent effects. These results suggest partial functional specialization within the class 3 lipase family and highlight the potential functional relevance of non‐patatin lipases in microalgal TAG metabolism.

## AUTHOR CONTRIBUTIONS

**Masako Iwai:** Conceptualization (equal); data curation (equal); investigation (equal); methodology (equal); writing – original draft (equal); writing – review and editing (equal). **Koichi Hori:** Data curation (equal); formal analysis (equal); methodology (equal); writing – review and editing (equal). **Tomokazu Kurita:** Methodology (equal); resources (equal); writing – review and editing (equal). **Kumiko Okazaki:** Writing – review and editing (equal). **Shinichiro Maeda:** Writing – review and editing (equal). **Akihide Takami:** Writing – review and editing (equal). **Takashi Yamamoto:** Funding acquisition (equal); writing – review and editing (equal). **Atsushi Sakamoto:** Funding acquisition (equal); writing – review and editing (equal). **Hiroyuki Ohta:** Conceptualization (equal); funding acquisition (equal); supervision (equal); writing – review and editing (equal). **Mie Shimojima:** Funding acquisition (equal); writing – review and editing (equal).

## FUNDING INFORMATION

This work was supported in part by the Japan Science and Technology Agency (JST) programs on Open Innovation Platform with Enterprises, Research Institute and Academia (OPERA, JPMJOP1613) and Open Innovation Platforms for Industry–academia Co‐creation (COI‐NEXT, JPMJPF2010).

## Supporting information


**Figure S1.** TALEN target site and sequences of gene‐disruption strains. a, TALEN target site for *No3LIP14* gene disruption. Black‐shaded boxes indicate the TALEN‐binding sites for the *No3LIP14* gene. Underlining indicates the exon region of the *No3LIP14* gene. The boldface *“ATG”* indicates the start codon. b, Sequences of *No3LIP14* gene‐disruption strains. Four genome‐edited strains were constructed using all‐in‐one TALEN vectors for targeting the *No3LIP14* gene. Underlining indicates TALEN‐binding sites. WT, wild type. c, TALEN target site for the *No3LIP7* gene disruption. Black‐shaded boxes indicate the TALEN target sites. Underlining indicates the exon region of *No3LIP7*. The boldface *“ATG”* indicates the start codon. d, Sequences of *No3LIP7* gene‐disruption strains. e, Sequences of isolated strains 3 and 4, in which *No3LIP7* was disrupted. f, Sequences of isolated strains 3–12, in which the *No3LIP7* gene was disrupted.
**Figure S2.** *Nannochloropsis* mutants generated by homologous recombination. (a, c, e) Schematic diagrams for genomic sequences of wild‐type (WT) and knockout (KO) strains. Boxes indicate exons, and diagonal hatching indicates sequences encoding lipase 3 domains. Regions between the sequences denoted by “Left Flank” and “Right Flank” were substituted with resistance‐gene cassettes for paromomycin (a, c) or zeocin (e), as indicated by the gray boxes. Left‐ and right‐facing arrowheads indicate primers used for genotyping PCR (b, d, f).
**Figure S3.** Domain architecture of NoLIP3 proteins. Pfam domains are shown as colored boxes. Predicted signal peptides identified by TargetP2 are indicated by “SP” and red boxes. Transmembrane helices predicted by TMHMM are shown as red underlines. The conserved GxSxG motif (containing the catalytic serine residue) is marked by red triangles. Putative catalytic triad residues are indicated as follows: Asp by blue triangles and His by green triangles.
**Figure S4.** Phylogenetic tree of orthogroup OG0000141 and bacterial lipase 3 homologs. The tree was constructed using the maximum‐likelihood method with the LG + G (5 categories) + I model. Bootstrap support values were estimated with the adaptive bootstrap method. The scale bar indicates 0.5 amino‐acid substitutions per site. Thick branches indicate nodes with identical topologies in both the maximum‐likelihood and neighbor‐joining trees. The abbreviations for species are as follows: Cme (*Cyanidioschyzon merolae* 10D), Ccr (*Chondrus crispus* Stackhouse), Cre (*Chlamydomonas reinhardtii* CC‐503), Kn (*Klebsormidium nitens* NIES‐2285), At (*Arabidopsis thaliana*), Esi (*Ectocarpus siliculosus* Ec32), Ptr (*Phaeodactylum tricornutum* CCAP 1055/1), Ehu (*Emiliania huxleyi* CCMP1516), and Gth (*Guillardia theta* CCMP2712) (Supplemental Table S1). The GenBank accession number or protein ID is indicated for each sequence.
**Figure S5.** Cell density and TAG content of wild type and the *No3LIP7* mutant under control or phosphate‐deficient conditions. Time course for each of cell density (a), TAG content per culture (b), and TAG content per cell (c) was monitored in control (left column) and –P (right column) medium. WT, wild type. Data represent the mean ± SD of four biological replicates. Each asterisk indicates a significant difference from wild type based on Dunnett's test (*p* < 0.05).
**Figure S6.** Cell density and TAG content of wild type and the *No3LIP10* mutant under phosphate‐deficient conditions. Time course for each of cell density (a), TAG content per culture (b), and TAG content per cell (c) was monitored in –P medium. WT, wild type. Data represent the mean ± SD of four biological replicates. Each asterisk indicates a significant difference from wild type based on Dunnett's test (*p* < 0.05).
**Figure S7.** Changes in TAG content of wild type and the *No3LIP18* mutant under phosphate‐deficient conditions. TAG content per culture (a) and TAG content per cell (b) were monitored in –P medium for the indicated number of days. WT, wild type. Data represent the mean ± SD of four biological replicates. Each asterisk indicates a significant difference from wild type based on Dunnett's test (*p* < 0.05).
**Figure S8.** Analysis of fatty‐acid composition of the TAG fractions isolated from the wild type and the *No3LIP6* and *No3LIP10* mutants. The TAG fractions were isolated from cells cultured in –P medium for the indicated number of days. WT, wild type. Data represent the mean ± SD of four biological replicates. Each asterisk indicates a significant difference from wild type based on Dunnett's test (*p* < 0.05).
**Figure S9.** Analysis of fatty‐acid composition of the TAG fractions isolated from the wild type and the *No3LIP7* mutant. The TAG fractions were isolated from cells cultured in control (left column) or –P (right column) medium for the indicated number of days. WT, wild type. Data represent the mean ± SD of four biological replicates. Each asterisk indicates a significant difference from wild type based on Dunnett's test (*p* < 0.05).
**Figure S10.** Analysis of fatty‐acid composition of the TAG fractions isolated from the wild type and the *No3LIP18* mutant. The TAG fractions were isolated from cells cultured in –P medium for the indicated number of days. WT, wild type. Data represent the mean ± SD of four biological replicates. Each asterisk indicates a significant difference from wild type based on Dunnett's test (*p* < 0.05).

**Table S1.** List of species used for ortholog analysis.
**Table S2.** List of 23 genes containing the Lipase_3 domain in *Nannochloropsis oceanica* NIES‐2145.
**Table S3.** Primers used in this study.
**Table S4.** Comparison of *Nannochloropsis oceanica* NIES‐2145 genome with *N. oceanica* CCMP1779 and *N. oceanica* IMET1.
**Appendix S1.** Supplementary methods.

## Data Availability

The data sets generated and/or analyzed during the current study are available as follows. The scaffolds and predicted genes of *N. oceanica* NIES‐2145 were deposited into DDBJ (Genome accession BAAHMV000000000). Raw RNA‐seq data were deposited into the DDBJ Sequence Read Archive (run accession DRR050373 [0 day], DRR658174 [3 days control], DRR658175 [3 days of nitrogen deficiency], DRR658176 [3 days of phosphate deficiency], DRR658177 [5 days control], DRR658178 [5 days of nitrogen deficiency], and DRR658179 [5 days of phosphate deficiency]). The origin of the data set used for ortholog analysis is described in the supplemental methods.
