# Supplementary material for: Identification and functional analysis of class 3 lipases associated with triacylglycerol metabolism in Nannochloropsis oceanica (Eustigmatophyceae)
Source: J Phycol. 2026 Jul 9;62(5):1478–93. doi: 10.1111/jpy.70203 (PMC13659388; doi:10.1111/jpy.70203)
Supplement: Supplementary file 1 [file JPY-62-1478-s002.docx]

**Supplementary Information**

**Supplemental methods**

**Genome sequencing**

Cultured *Nannochloropsis oceanica* NIES-2145 cells were frozen in liquid nitrogen and ground to a fine powder with a mortar with 4 volumes of Buffer AP1 (QIAGEN DNeasy Plant Mini kit). DNA was subsequently extracted according to the DNeasy Plant Handbook. The purified DNA was sequenced using the Roche 454 GS FLX Titanium platform, generating 1,297,258 single-end reads and 1,457,052 mate-pair reads (3-kbp inserts). Additional sequencing was performed on the Illumina GAIIx platform, yielding 144,608,883 paired 101-bp reads. The sequencing data are available in the DDBJ Sequence Read Archive (BioProject Accession PRJDB20440, Run Accession DRR658168, DRR658169, DRR658170, DRR658171, DRR658172).

**Genomic DNA assembly**

Illumina paired-end reads were assembled using SOAPdenovo 2 (r240, k-mer size of 73) (Luo et al. 2012), producing 324,220 contigs. Of these, 319,870 contigs shorter than 2000 bp and 454 GS FLX Titanium reads were assembled using Newbler 2.8 (Margulies et al. 2005), resulting in 202 scaffolds (N50 = 559,539 bp, total length = 30,221,314 bp). These scaffolds were assembled using minimus2 (Sommer et al. 2007), together with 4350 SOAPdenovo2 contigs longer than 2000 bp, yielding 145 scaffolds (N50 = 559,529 bp, total length = 29,924,532 bp).

**Gene prediction**

RNA-seq reads of *N. oceanica* NIES-2145 (DRR658173) were aligned to the assembled scaffolds using tophat2 v2.0.9 (Kim et al. 2013) to support gene prediction. The alignments were used to generate intron hint files for AUGUSTUS 3.0.1 (Stanke et al. 2008). A manually curated set of 488 gene models was used to train AUGUSTUS, which subsequently predicted 8617 genes with transcript evidence. The predicted genes and scaffolds were deposited in DDBJ (Genome accession BAAHMV000000000). Genome assembly and gene prediction status were compared with those of the *N. oceanica* CCMP1779 v2.0 and *N. oceanica* IMET1v2 genomes (Table S4).

**Supplemental References**

Bowler, C., Allen, A.E., Badger, J.H., Grimwood, J., Jabbari, K., Kuo, A., Maheswari, U. et al. (2008). The *Phaeodactylum* genome reveals the evolutionary history of diatom genomes. *Nature*, 456(7219), 239–244.

Cock, J.M., Sterck, L., Rouzé, P., Scornet, D., Allen, A.E., Amoutzias, G., Anthouard, V. et al. (2010). The Ectocarpus genome and the independent evolution of multicellularity in brown algae. *Nature*, 465(7298), 617–621.

Collén, J., Porcel, B., Carré, W., Ball, S.G., Chaparro, C., Tonon, T., Barbeyron, T. et al. (2013). Genome structure and metabolic features in the red seaweed *Chondrus crispus* shed light on evolution of the Archaeplastida. *Proceedings of the National Academy of Sciences of the United States of America*, 110(13), 5247–5252.

Curtis, B.A., Tanifuji, G., Burki, F., Gruber, A., Irimia, M., Maruyama, S., Arias, M.C. et al. (2012). Algal genomes reveal evolutionary mosaicism and the fate of nucleomorphs. *Nature*, 492(7427), 59–65.

Gong, Y., Kang, N.K., Kim, Y.U., Wang, Z., Wei, L., Xin, Y., Shen, C. et al. (2020). The NanDeSyn database for *Nannochloropsis* systems and synthetic biology. *The Plant Journal: For Cell and Molecular Biology*, 104(6), 1736–1745.

Hori, K., Maruyama, F., Fujisawa, T., Togashi, T., Yamamoto, N., Seo, M., Sato, S. et al. (2014). *Klebsormidium flaccidum* genome reveals primary factors for plant terrestrial adaptation. *Nature Communications*, 5(1), 3978.

Kim, D., Pertea, G., Trapnell, C., Pimentel, H., Kelley, R. & Salzberg, S.L. (2013). TopHat2: accurate alignment of transcriptomes in the presence of insertions, deletions and gene fusions. *Genome Biology*, 14(4), R36.

Lamesch, P., Berardini, T.Z., Li, D., Swarbreck, D., Wilks, C., Sasidharan, R., Muller, R. et al. (2012). The Arabidopsis Information Resource (TAIR): improved gene annotation and new tools. *Nucleic Acids Research*, 40(Database issue), D1202-1210.

Luo, R., Liu, B., Xie, Y., Li, Z., Huang, W., Yuan, J., He, G. et al. (2012). SOAPdenovo2: an empirically improved memory-efficient short-read de novo assembler. *GigaScience*, 1(1), 18.

Manni, M., Berkeley, M.R., Seppey, M., Simão, F.A. & Zdobnov, E.M. (2021). BUSCO Update: Novel and Streamlined Workflows along with Broader and Deeper Phylogenetic Coverage for Scoring of Eukaryotic, Prokaryotic, and Viral Genomes. *Molecular Biology and Evolution*, 38(10), 4647–4654.

Margulies, M., Egholm, M., Altman, W.E., Attiya, S., Bader, J.S., Bemben, L.A., Berka, J. et al. (2005). Genome sequencing in microfabricated high-density picolitre reactors. *Nature*, 437(7057), 376–380.

Matsuzaki, M., Misumi, O., Shin-I, T., Maruyama, S., Takahara, M., Miyagishima, S.-Y., Mori, T. et al. (2004). Genome sequence of the ultrasmall unicellular red alga *Cyanidioschyzon merolae* 10D. *Nature*, 428(6983), 653–657.

Meier-Kolthoff, J.P., Carbasse, J.S., Peinado-Olarte, R.L. & Göker, M. (2022). TYGS and LPSN: a database tandem for fast and reliable genome-based classification and nomenclature of prokaryotes. *Nucleic Acids Research*, 50(D1), D801–D807.

Merchant, S.S., Prochnik, S.E., Vallon, O., Harris, E.H., Karpowicz, S.J., Witman, G.B., Terry, A. et al. (2007). The Chlamydomonas Genome Reveals the Evolution of Key Animal and Plant Functions. *Science (New York, N.Y.)*, 318(5848), 245–250.

Read, B.A., Kegel, J., Klute, M.J., Kuo, A., Lefebvre, S.C., Maumus, F., Mayer, C. et al. (2013). Pan genome of the phytoplankton Emiliania underpins its global distribution. *Nature*, 499(7457), 209–213.

Sommer, D.D., Delcher, A.L., Salzberg, S.L. & Pop, M. (2007). Minimus: a fast, lightweight genome assembler. *BMC Bioinformatics*, 8(1), 64.

Stanke, M., Diekhans, M., Baertsch, R. & Haussler, D. (2008). Using native and syntenically mapped cDNA alignments to improve de novo gene finding. *Bioinformatics (Oxford, England)*, 24(5), 637–644.

**Table S1**

List of species used for ortholog analysis

| Group |  |  | Scientific name | Abbreviation | Data Resource  Supplemental Reference |
| --- | --- | --- | --- | --- | --- |
| Archaeplastida | | | | | |
|  | Red algae | | | | |
|  |  |  | *Cyanidioschyzon merolae* 10D | Cme | project site (Matsuzaki et al. 2004) |
|  |  |  | *Chondrus crispus* Stackhouse | Ccr | JGI (Collén et al. 2013) |
|  | Green algae | | | | |
|  |  |  | *Chlamydomonas reinhardtii* CC-503 | Cre | JGI v5.6 (Merchant et al. 2007) |
|  | Streptophyte algae | | | | |
|  |  |  | *Klebsormidium nitens* NIES-2285 | Kn | project site v1.1(Hori et al. 2014) |
|  | Embryophyte | | | | |
|  |  |  | *Arabidopsis thaliana* | At | TAIR10 (Lamesch et al. 2012) |
| Secondary endosymbiotic algae | | | | | |
|  | Stramenopiles Ochrophyta | | | | |
|  |  | Phaeophyceae | | | |
|  |  |  | *Ectocarpus siliculosus* Ec 32 | Esi | JGI (Cock et al. 2010) |
|  |  | Bacillariophyceae | | | |
|  |  |  | *Phaeodactylum tricornutum* CCAP 1055/1 | Ptr | JGI v2.0 (Bowler et al. 2008) |
|  |  | Eustigmatophytes | | | |
|  |  |  | *Nannochloropsis oceanica* NIES-2145 | Noc | This study |
|  | Haptophyte | | | | |
|  |  |  | *Emiliania huxleyi* CCMP1516 v1.0 | Ehu | JGI (Read et al. 2013) |
|  | Cryptomonad | | | | |
|  |  |  | *Guillardia theta* CCMP2712 v1.0 | Gth | JGI (Curtis et al. 2012) |

**Table S2**

List of 23 genes containing the Lipase_3 domain in *N. oceanica* NIES-2145

| Orthogroup | LIP No. | Locus ID | Protein ID |
| --- | --- | --- | --- |
| OG0000141 | No3LIP1 | NocV09_00200110 | GAB5029874.1 |
| OG0000141 | No3LIP3 | NocV09_00400910 | GAB5030815.1 |
| OG0000141 | No3LIP5 | NocV09_00802570 | GAB5032291.1 |
| OG0000141 | No3LIP7 | NocV09_00901180 | GAB5032425.1 |
| OG0000141 | No3LIP9 | NocV09_01201880 | GAB5033236.1 |
| OG0000141 | No3LIP11 | NocV09_01300170 | GAB5033275.1 |
| OG0000141 | No3LIP15 | NocV09_02800460 | GAB5035625.1 |
| OG0000141 | No3LIP18 | NocV09_03300750 | GAB5036094.1 |
| OG0000141 | No3LIP21 | NocV09_05600180 | GAB5036986.1 |
| OG0000141 | No3LIP22 | NocV09_07400050 | GAB5037350.1 |
| OG0000178 | No3LIP12 | NocV09_01300810 | GAB5033340.1 |
| OG0000178 | No3LIP13 | NocV09_02101390 | GAB5034835.1 |
| OG0000341 | No3LIP8 | NocV09_01200890 | GAB5033135.1 |
| OG0000746 | No3LIP17 | NocV09_03100650 | GAB5035929.1 |
| OG0003312 | No3LIP4 | NocV09_00702430 | GAB5031981.1 |
| OG0003312 | No3LIP6 | NocV09_00901140 | GAB5032421.1 |
| OG0003312 | No3LIP10 | NocV09_01300050 | GAB5033260.1 |
| OG0003312 | No3LIP14 | NocV09_02600380 | GAB5035396.1 |
| OG0003312 | No3LIP16 | NocV09_03000300 | GAB5035818.1 |
| OG0003312 | No3LIP19 | NocV09_03900440 | GAB5036442.1\|GAB5036443.1 |
| OG0003312 | No3LIP20 | NocV09_03900450 | GAB5036444.1 |
| OG0003312 | No3LIP23 | NocV09_12300050 | GAB5037912.1 |
| OG0051624 | No3LIP2 | NocV09_00201200 | GAB5029986.1 |

**Table S3**

Primers used in this study

| **Use** | **name** | **Forward primer (5´–3´)** |
| --- | --- | --- |
|  |  | **Reverse primer (5´–3´)** |
| *No3LIP14* knockout construction | LIP14-F | GCACTCGGTAATGGCTGTCTACACG |
|  | LIP14-R | ATTGTGCTGTGAACTGCTCCTCTCG |
| *No3LIP7* knockout construction | LIP7-1-F | GATCGACCAGAACAGAAGACCATGC |
|  | LIP7-1-R | TCATCAGATTGGAAGCCAAGGTCAG |
| *No3LIP6* knockout construction | LIP6-LF-F | CTGGTAGATGGGCAGGTGTGAG |
| (to amplify the 5**´**-flanking region) | LIP6-LF-R | AAGTTAACACAACGAAGCGCCG |
| *No3LIP6* knockout construction | LIP6-RF-F | GCCTGACTTGCCCCAATCCTAC |
| (to amplify the 3**´**-flanking region) | LIP6-RF-R | GGAACAGGAGCTTCATATTC |
| *No3LIP10* knockout construction | LIP10-LF-F | GGTGAGATAATGGGGCAAATGC |
| (to amplify the 5**´**-flanking region) | LIP10-LF-R | GGTCTTGGCGCCTCCGTTTGCG |
| *No3LIP10* knockout construction | LIP10-RF-F | TGGAGCCCGGACGTTTAGAGAC |
| (to amplify the 3**´**-flanking region) | LIP10-RF-R | CACCTCTTCATCTCAGGTTGACC |
| *No3LIP18* knockout construction | LIP18-LF-F | GGTTGACCAAAGTGTCCGAGG |
| (to amplify the 5**´**-flanking region) | LIP18-LF-R | TGCCGGACTCGAGGAGGCTCCTGAT |
| *No3LIP18* knockout construction | LIP18-RF-F | GAAAGAGGAGAAACAGACTCGC |
| (to amplify the 3**´**-flanking region) | LIP18-RF-R | AGACCTATAATCTCACTGCTCC |
| PCR genotyping for *No3LIP6* knockout | a | TACTACTACTGCTACCGACTGATC |
|  | b | CATTTCTTCGGCATAGAAGGGGAC |
| PCR genotyping for *No3LIP6* knockout | c | GCCTTTCCAGAGATAGAGATGG |
|  | d | TGTCGACGTTGGTTTCGAGC |
| PCR genotyping for *No3LIP10* knockout | e | GAGTGACGGGCACTTTGAGA |
|  | f | TTCCGCAGAAGAAGACCACC |
| PCR genotyping for *No3LIP10* knockout | g | CTGGGGCCGATCTCCAAATTCG |
|  | h | AGGGGAGCTTGAGGCAGCATGG |
| PCR genotyping for *No3LIP18* knockout | i | CCAAAATGTTCTTCCCACCC |
|  | j | CACTCCGCTTCCAAAAAC |
| PCR genotyping for *No3LIP18* knockout | k | CGAGCAATGCGGAGAGCCCT |
|  | l | AGGGGAGCTTGAGGCAGCATGG |

**Table S4**

Comparison of *N. oceanica* NIES-2145 genome with *N. oceanica* CCMP1779 and *N. oceanica* IMET1

|  | NIES-2145  (This study) | CCMP1779 v2.0  ( JGI, https://mycocosm.jgi.doe.gov/Nanoce1779_2/Nanoce1779_2.home.html ) | IMET1v2  (Gong et al. 2020) |
| --- | --- | --- | --- |
| Total length | 29,924,532 | 36,454,932 | 31,002,849 |
| GC contents | 54.1 | 54.4 | 54.3 |
| Number of Scaffolds | 145 | 305 | 30 (chromosome) |
| N50 | 559,529 | 664,048 | 1,168,160 |
| L50 | 16 | 17 | 11 |
| Genome-to-Genome Distance Calculator 3.0 Formula 2 DDH  (Meier-Kolthoff et al. 2022) | Reference:CCMP1779v2.0  94.9%  Reference IMET1v2  97.1% | Reference:NIES-2145  94.9%  Reference IMET1v2  95.0% | Reference:NIES-2145  97.1%  Reference:CCMP1779v2.0  95.0% |
| Number of Predicted mRNA | 8,357 | 10,641 | 12,442 |
| Complete BUSCO genes  v5.8.0 (Manni et al. 2021) | 80.4% | 74.1% | 84.7% |
| Number of Lipase_3 containing genes | 23 | 24 | 24 |

**Supplementary Figure legends**

**Figure S1 TALEN target site and** **sequences of gene-disruption strains.**

**a,** TALEN target site for *No3LIP14* disruption. Black-shaded boxes indicate the TALEN-binding sites for *No3LIP14*. Underlining indicates the exon region of *No3LIP14.* The boldface *“ATG”* indicates the start codon. **b,** Sequences of *No3LIP14* gene-disruption strains. Four genome-edited strains were constructed using all-in-one TALEN vectors for targeting *No3LIP14*. Underlining indicates TALEN-binding sites. WT, wild type. **c,** TALEN target site for *No3LIP7* disruption. Black-shaded boxes indicate the TALEN target sites. Underlining indicates the exon region of *No3LIP7.* The boldface *“ATG”* indicates the start codon. **d,** Sequences of *No3LIP7* gene-disruption strains. **e,** Sequences of isolated strains 3 and 4, in which *No3LIP7* was disrupted. **f,** Sequences of isolated strains 3­–12, in which *No3LIP7* was disrupted.

**Figure S2 *Nannochloropsis* mutants generated by homologous recombination.**

(a, c, e) Schematic diagrams for genomic sequences of wild-type (WT) and knockout (KO) strains. Boxes indicate exons, and diagonal hatching indicates sequences encoding lipase 3 domains. Regions between the sequences denoted by “Left Flank” and “Right Flank” were substituted with resistance-gene cassettes for paromomycin (a, c) or zeocin (e), as indicated by the gray boxes. Left- and right-facing arrowheads indicate primers used for genotyping PCR (b, d, f).

**Figure S3 Domain architecture of NoLIP3 proteins.**

Pfam domains are shown as colored boxes. Predicted signal peptides identified by TargetP2 are indicated by “SP” and red boxes. Transmembrane helices predicted by TMHMM are shown as red underlines. The conserved GxSxG motif (containing the catalytic serine residue) is marked by red triangles. Putative catalytic triad residues are indicated as follows: Asp by blue triangles and His by green triangles.

**Figure S4 Phylogenetic tree of orthogroup OG0000141 and bacterial lipase 3 homologs.**

The tree was constructed using the maximum-likelihood method with the LG+G (5 categories) +I model. Bootstrap support values were estimated with the adaptive bootstrap method. The scale bar indicates 0.5 amino-acid substitutions per site. Thick branches indicate nodes with identical topologies in both the maximum-likelihood and neighbor-joining trees. The abbreviations for species are as follows: Cme (*Cyanidioschyzon merolae* 10D), Ccr (*Chondrus crispus* Stackhouse), Cre (*Chlamydomonas reinhardtii* CC-503), Kn (*Klebsormidium nitens* NIES-2285), At (*Arabidopsis thaliana*), Esi (*Ectocarpus siliculosus* Ec32), Ptr (*Phaeodactylum tricornutum* CCAP 1055/1), Ehu (*Emiliania huxleyi* CCMP1516), and Gth (*Guillardia theta* CCMP2712) (Supplemental Table S1). The GenBank accession number or protein ID is indicated for each sequence.

**Figure S5 Cell density and TAG content of wild type and *No3LIP7* under control or phosphate-deficient conditions.**

Time course for each of cell density (a), TAG content per culture (b), and TAG content per cell (c) was monitored in control (left column) and –P (right column) medium. WT, wild type. Data represent the mean ± S.D. of four biological replicates. Each asterisk indicates a significant difference from wild type based on Dunnett’s test (*P* < 0.05).

**Figure S6 Cell density and TAG content of wild type and *No3LIP10* under phosphate-deficient conditions.**

Time course for each of cell density (a), TAG content per culture (b), and TAG content per cell (c) was monitored in –P medium. WT, wild type. Data represent the mean ± S.D. of four biological replicates. Each asterisk indicates a significant difference from wild type based on Dunnett’s test (*P* < 0.05).

**Figure S7 Changes in TAG content of wild type and *No3LIP18* under phosphate-deficient conditions.**

TAG content per culture (a) and TAG content per cell (b) were monitored in –P medium for the indicated number of days. WT, wild type. Data represent the mean ± S.D. of four biological replicates. Each asterisk indicates a significant difference from wild type based on Dunnett’s test (*P* < 0.05).

**Figure S8 Analysis of fatty-acid composition of the TAG fractions isolated from wild type, *No3LIP6* and *No3LIP10*.**

The TAG fractions were isolated from cells cultured in –P medium for the indicated number of days. WT, wild type. Data represent the mean ± S.D. of four biological replicates. Each asterisk indicates a significant difference from wild type based on Dunnett’s test (*P* < 0.05).

**Figure S9 Analysis of fatty-acid composition of the TAG fractions isolated from wild type and *No3LIP7*.**

The TAG fractions were isolated from cells cultured in control (left column) or –P (right column) medium for the indicated number of days. WT, wild type. Data represent the mean ± S.D. of four biological replicates. Each asterisk indicates a significant difference from wild type based on Dunnett’s test (*P* < 0.05).

**Figure S10 Analysis of fatty-acid composition of the TAG fractions isolated from wild type and *No3LIP18*.**

The TAG fractions were isolated from cells cultured in –P medium for the indicated number of days. WT, wild type. Data represent the mean ± S.D. of four biological replicates. Each asterisk indicates a significant difference from wild type based on Dunnett’s test (*P* < 0.05).
